# Helicopter Test of a Strapdown Airborne Gravimetry System

**DOI:** 10.3390/s18093121

**Published:** 2018-09-16

**Authors:** Tim Enzlberger Jensen, Rene Forsberg

**Affiliations:** National Space Institute, Technical University of Denmark, 2800 Kgs. Lyngby, Denmark; rf@space.dtu.dk

**Keywords:** airborne gravimetry, strapdown inertial measurement unit, helicopter test, Kalman filter

## Abstract

Airborne gravimetry from a helicopter has been a feasible tool since the 1990s, with gravimeters mounted on a gyro-stabilised platform. In contrast to fixed-wing aircrafts, the helicopter allows for a higher spatial resolution, since it can move slower and closer to the ground. In August 2016, a strapdown gravimetry test was carried out over the Jakobshavn Glacier in Greenland. To our knowledge, this was the first time that a strapdown system was used in a helicopter. The strapdown configuration is appealing because it is easily installed and requires no operation during flight. While providing additional information over the thickest part of the glacier, the survey was designed to assess repeatability both within the survey and with respect to profiles flown previously using a gyro-stabilised gravimeter. The system’s ability to fly at an altitude following the terrain, i.e., draped flying, was also tested. The accuracy of the gravity profiles was estimated to 2 mGal and a method for inferring the spatial resolution was investigated, yielding a half-wavelength spatial resolution of 4.5 km at normal cruise speed.

## 1. Introduction

The first airborne gravimetry flight test was carried out in 1958 by mounting a LaCoste shipboard gravimeter on board an Air Force KC-135 fixed-wing aircraft [1]. The observed gravity values were averaged over 5 min intervals, yielding an accuracy of about 10 mGal, which was adequate for geodetic purposes at the time. However, since this accuracy did not meet the requirements of the exploration industry, the idea of using a helicopter platform emerged. The helicopter would be able to fly closer to the ground and move at a slower speed, allowing for a higher spatial resolution of the gravity measurements. After an unsuccessful test in 1963, the U.S. Naval Oceanographic Office made the first successful helicopter test in 1965 using an Air Force CH3E helicopter, equipped with the latest LaCoste & Romberg platform stabilised gravimeter [2].

Initially, most development of airborne gravimetry was driven by government mapping agencies and military branches of the U.S. government. These efforts were aimed at surveying large-scale regions and involved fixed-wing aircrafts [3,4]. From 1970, the exploration industry joined the effort, leading to the introduction of a helicopter-borne gravity system by Carson Services in 1977 and other companies in the 1990s [5]. Most airborne systems consist of a single-axis accelerometer on a gyro-stabilised platform that attempts to null the horizontal accelerations through a mechanical feedback loop. The AIRGrav system from Sander Geophysics (SGL) differs from these systems in that it uses three inertial-grade accelerometers on a fully gyro-stabilised platform, which does not attempt to compensate for the horizontal accelerations. The advantages of this approach are: lower accelerometer noise, higher resolution and less sensitivity to turbulence [6]. The claimed resolution of the AIRGrav system is 0.2 mGal at 2.2 km (fixed-wing) and 0.2 mGal at 0.7 km (helicopter). Although helicopter-mounted gravimetry is mostly aimed at exploration, it is also carried out for research purposes such as studying tectonically active regions [7,8] and mapping bathymetry below ice shelves [9]. In particular, the use of helicopter-based gravimetry operated from a ship at sea may play an important role in studying ice shelves and marine-terminating glaciers in remote areas otherwise inaccessible.

The AIRGrav system development was to some degree inspired by pioneering work on the use of Inertial Measurement Units (IMU) for airborne gravimetry at the University of Calgary in the 1990s [10]. Since the gyroscopes of the IMU measures angular rates, the rotational motion can be accounted for computationally, instead of having a control loop feeding a mechanical platform. As a result, the platform can be completely neglected and the IMU installed in a strapdown configuration, i.e., by physically attaching the unit to the vehicle. This approach is appealing because it is easily installed and does not require any operation during the flight. The first test of a Strapdown Airborne Gravimetry (SAG) system was carried out in 1995 using a fixed-wing aircraft. The reported accuracy was 2–3 mGal at 5–7 km (half wavelength) resolution after applying 90–120 s (full wavelength) filtering [11]. The higher dynamic range of the IMU made the gravity system more resilient to turbulence and less filtering was required to obtain an accuracy similar to the traditional single-sensor stabilised-platform systems [12]. However, the instability of the accelerometers propagates into the gravity estimates and corrupts the long wavelength information, which is vital for geodetic applications. The majority of the sensor instability has been shown to originate from temperature variation and to a large extent accounted for using laboratory calibration methods [13]. In collaboration with the Technical University of Darmstadt, the National Space Institute of Denmark (DTU Space) has flown a SAG system on a number of campaigns showing significant improvement after applying temperature calibrations [14]. A large component of the difference in accelerometer stability between the IMU and traditional systems may therefore be due to temperature stabilisation.

In order to test the feasibility of using a strapdown IMU in a helicopter environment, a flight test was carried out in August 2016 over the Jakobshavn Glacier in Greenland. This location was chosen because it has previously been surveyed using the SGL AIRGrav system within NASA’s Operation IceBridge (OIB). Since the fast-flowing ice stream is more than 1 km thick [15], the relatively fast-moving fixed-wing aircraft flown by OIB is not expected to capture the full resolution of the gravity signal in this area. The slower-moving helicopter platform may therefore add information to the gravity signal over the glacier, while the OIB flight lines provide a reference for the helicopter estimates. It should be noted that Jakobshavn Glacier was surveyed by a helicopter in 2012 using the SGL AIRGrav system [16]. This data was however unavailable to the authors for this study.

## 2. Instrumentation, Survey Overview and Data

**Figure 1:**
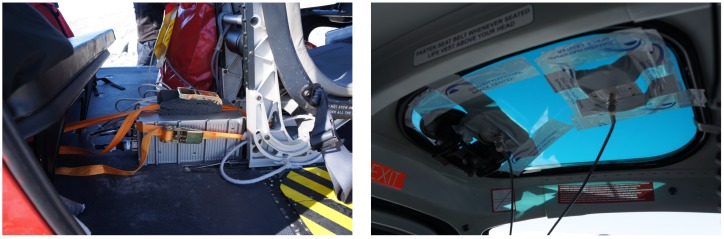
Photographs from the installation: (**left**) the Inertial Measurement Unit (IMU) is physically strapped to the floor of the helicopter; (**right**) the GNSS antenna is taped to the inside of the windscreen.

In August 2016, a SAG system was mounted inside a Eurocopter AS350 helicopter at Ilulissat airport (JAV), Greenland. The SAG system consists of an iMAR iNAT IMU unit (iNAT), two JAVAD DELTA GNSS receivers and two NovAtel ANT-532-C dual frequency GNSS antennas along with some batteries and cables. The installation was done using straps and tape and took less than an hour, see Figure 1.

**Figure 2:**
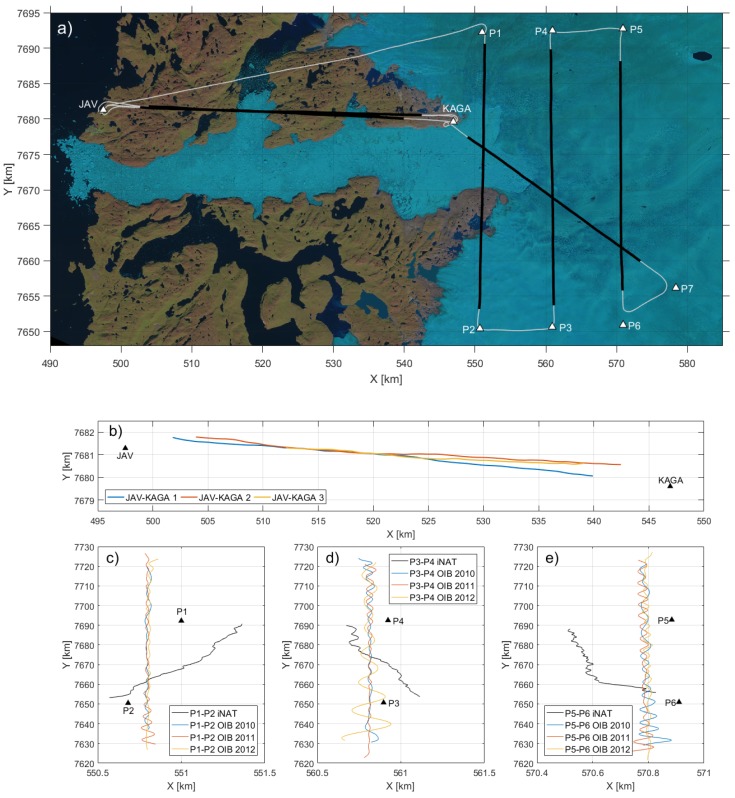
Survey overview: (**a**) ground track of the entire survey (white) along with line profiles (black) on top of Landsat-8 image from 9 August 2016; (**b**) ground track of the three repeat lines between Ilulissat Airport (JAV) and Kangia North GPS station (KAGA); (**c**) ground track of the profiles between P1 and P2; (**d**) ground track of the profiles between P3 and P4; (**e**) ground track of the profiles between P5 and P6. Coordinates are with respect to UTM zone 22. Notice the difference in scale between the *x*- and *y*-axes.

The entire survey amounts to approximately 400 km (3.5 h), see Figure 2, and was designed to repeat flight lines both from OIB and within the survey itself. The profile between JAV and Kangia North GPS station (KAGA) was repeated three times, but not flown at the same altitude for operational reasons. The three profiles between the waypoints P1–P2, P3–P4 and P5–P6 correspond to flight lines from OIB. Two of these lines were flown in draped mode and one at constant altitude. From Figure 2a–c, it is evident that the fixed-wing aircraft from OIB was equipped with an autopilot, whereas the helicopter was not. Finally, a line from P7 to KAGA that crosses over the three OIB lines was additionally flown. This resulted in seven flight lines of approximately 300 km (1.5 h). The average ground speed for these seven profiles was 52 m/s (standard deviation of 6 m/s).

### 2.1. The IMU Data

The iNAT unit outputs accelerations and angular rates at 300 Hz with associated time and temperature stamps. It contains an internal GNSS receiver that provides time stamps in GPS time. A simple warm-up temperature calibration was applied to the vertical *z*-axis accelerometer only [17]. This led to a reduction in the accelerometer drift (see Table 1).

### 2.2. The GNSS Data

The GNSS receivers log both GPS and GLONASS observations at 1 Hz. Using NovAtel’s Waypoint software suite, a Precise Point Positioning (PPP) solution was produced using the final GPS and GLONASS ephemerides from the International GNSS Service (IGS). The GNSS observations were thus processed independently from the IMU observations, in order to arrive at position and velocity estimates, with associated error covariance matrices.

Using data from the nearby KAGA GPS station, a differential GNSS solution was also produced. The differential solution did, however, not lead to any improvement compared to the PPP solution. One possible explanation is that the KAGA receiver only logs GPS observations, leading to a reduction in the number of satellites used for the solution.

### 2.3. AIRGrav Gravity Estimates from Operation IceBridge

Gravity estimates from OIB are available with 70 s, 100 s and 140 s full wavelength filters applied to the Eötvös corrected gravity disturbance estimates (see acknowledgements). The three lines flown in this survey were covered by OIB in the years 2010, 2011 and 2012. A visual comparison of the three profiles for each line indicated that the 100 s filter led to reasonable agreement between the gravity estimates. The internal mean and standard deviation (STD) of the difference in OIB gravity estimates for each line and year are shown in Table 2. Since the gravity response associated with glacier mass change is expected to be below 1 mGal for this two year period, it is clear that the stated 0.2 mGal accuracy of the AIRGrav system is not reached over the Jakobshavn Glacier. This may be due to the high speed of the aircraft not accounting for the full resolution of the gravity signal in this area. The average ground speed for these nine profiles was 134 m/s (standard deviation of 9 m/s), meaning that the spatial resolution is approximately 6.7 km (half wavelength). The nine OIB lines were flown in draped mode at a similar altitude for each line (the maximum difference is 300 m and the standard deviation is 80 m).

## 3. Processing Methodology

Observations from IMU and GNSS are often combined in order to improve the navigation solution. Although the main objective is not improving the navigation solution, the tools for IMU/GNSS integration are well-developed and can be exploited here. In the first two of the following five sub-sections, the concepts of inertial navigation and IMU/GNSS integration will be briefly introduced. In the third sub-section, we describe how this framework can be exploited to arrive at gravity estimates, the fourth sub-section introduces external gravity observations, i.e., tie values, and the final sub-section outlines how smoothing is performed.

In this context, it is important to introduce some reference frames that are relevant for our purposes (see Table 3). Since the IMU is rigidly attached to the helicopter, the observations are naturally resolved about the body frame (*b*-frame) of the helicopter. Being inertial sensors, the accelerometers and gyroscopes measure with respect to inertial space, which is represented by an inertial reference frame (*i*-frame). The Earth-Centred-Earth-Fixed frame (*e*-frame) is relevant because coordinates and velocity are defined with respect to this frame. Finally, the attitude of the vehicle is usually specified with respect to the navigation frame (*n*-frame), which is moreover closely related to the gravity field.

### 3.1. Inertial Navigation

Initialisation of the position is done using the GNSS solution, while the attitude is initialised through levelling and gyrocompassing ([18] (Chapter 5.6.2)). After initialisation, inertial navigation is performed by sequentially adding small increments derived by integrating the IMU observations of specific force, $f^{b}$, and angular rate, $\omega_{ib}^{b}$. Before integration, the observations are transformed from the *b*-frame into the *n*-frame and corrected for the effect of gravity along with any fictitious forces arising from the choice of reference frame. This process is expressed mathematically in terms of a coupled set of differential equations ([19] (Chapter 4.3.4)):

(1) $$\begin{array}{cc} \dot{\phi } & =v_{N}/\left(R_{N}+h\right),\\ \dot{\lambda } & =v_{E}/\left(R_{E}+h\right)/\cos\phi ,\\ \dot{h} & =-v_{D},\\ {\dot{v}}^{n} & =C_{b}^{n}\;f^{b}-\left(2\Omega_{ie}^{n}+\Omega_{en}^{n}\right)\;v^{n}+g^{n},\\ {\dot{C}}_{b}^{n} & =C_{b}^{n}\;\Omega_{nb}^{b}\;, \end{array}$$

where $\left(\phi ,\lambda ,h\right)$ are geodetic coordinates, $v^{n}$ the Earth-referenced velocity resolved about the *n*-frame axes and $C_{b}^{n}$ is the rotation matrix from the *b*-frame to the *n*-frame. The parameters $R_{E}$ and $R_{N}$ are the radii of curvature of the prime vertical and meridian, respectively. The term $\Omega_{ie}^{n}$ is the rotation of the Earth with respect to inertial space and $\Omega_{en}^{n}$ is the transport-rate, i.e., the rotational motion required to keep the reference frame aligned with the north, east and vertical axes as the vehicle travels across the surface of the Earth. Both of these rotational rates are resolved about the *n*-frame axes and expressed in skew-symmetric form. For a more complete introduction, the reader is referred to standard textbooks, e.g., [18,19,20]. Finally, the rotational rate, $\Omega_{nb}^{b}$, is related to the observed angular rate as

(2) $$\omega_{nb}^{b}=\omega_{ne}^{b}+\omega_{ei}^{b}+\omega_{ib}^{b}=\omega_{ib}^{b}-C_{n}^{b}\;\left(\omega_{en}^{n}+C_{e}^{n}\;\omega_{ie}^{e}\right).\;$$

The gravity vector, $g^{n}$, is approximated using a model of normal gravity ([21] (Chapter 2.8)), which can be computed as described in ([22] (Chapter 4)). The implementation of Equation (1) is discussed in ([18] (Chapter 5)). The combined system of IMU and inertial navigation processor is denoted an Inertial Navigation System (INS). The attitude in terms of three Euler angles, $\psi_{nb}=\left(\alpha_{nb},\beta_{nb},\gamma_{nb}\right)$, can be derived from the transformation matrix, $C_{b}^{n}$, as ([18] (Equation (2.25))):

(3) $$\alpha_{nb}=\arctan_{2}\left(C_{b3,2}^{n},C_{b3,3}^{n}\right)\;,\;\beta_{nb}=-\arcsin\left(C_{b3,1}^{n}\right)\;\mathrm{and}\;\gamma_{nb}=\arctan_{2}\left(C_{b2,1}^{n},C_{b1,1}^{n}\right).\;$$

### 3.2. IMU/GNSS Integration

The GNSS position estimates are only used to initialise the position for inertial navigation, meaning that the INS and GNSS navigation solutions are independent. The GNSS system provides estimates of position and velocity, while the INS provides estimates of position, velocity and attitude. These two estimates are combined in a cascaded (loosely coupled) approach using an Empirical Kalman Filter (EKF). The Kalman filter framework revolves around a linear dynamic system model ([23] (Equations (4)–(102))):

(4) $$\dot{x}=F\left(t\right)\;x\left(t\right)+G\left(t\right)\;w_{s}\left(t\right),\;$$

where x(t) is known as the state vector and contains all the variables describing the system, i.e., position, velocity, attitude and sensor biases, F(t) is the system matrix describing the dynamics of the system, i.e., the navigation equations, $w_{s}\left(t\right)$ is a vector representing stochastic input to the system and G(t) is a system noise distribution matrix, relating the stochastic driving terms to the state variables. The stochastic components imply that the state variables also have a stochastic nature and are defined in terms of probability density functions (PDFs). However, assuming that the associated PDFs are Gaussian, these distributions are completely defined in terms of their first two moments, i.e., the mean and covariance.

The system matrix, F(t), is formed using the navigation Equation (1), while the system noise vector, $w_{s}\left(t\right)$, represents sensor errors such as random noise and bias variation. Since the system model is linear, the navigation equations are linearised about the trajectory generated by the INS. This kind of linearisation is what characterises the EKF. Inertial navigation is then performed in a recursive manner by generating a navigation solution from current time, $t_{k}$, until the time where the next GNSS estimates are available, $t_{k+1}$. The associated error covariance estimates are accordingly propagated as

(5) $$P_{k+1}=\Phi_{k}\;P_{k}\;\Phi_{k}^{⊤}+\Gamma_{k}\;Q_{k}\;\Gamma_{k}^{⊤},\;$$

where the transition matrix, $\Phi_{k}$, and system noise, $\Gamma_{k}\;Q_{k}\;\Gamma_{k}^{⊤}$, can be formed from Equation (4) using the method of Van Loan [24,25]. The transition matrix, $\Phi_{k}$, is formed using the system matrix, F(t), such that it eventually defines the correlation between the navigation parameters through the error covariance matrix. In this way, the stochastic driving terms do also not influence the navigation estimates themselves, but only the error covariance matrix. These stochastic processes are defined by the user and will eventually determine the weighting of information between INS and GNSS navigation estimates. Here, random noise on the accelerometer and gyro observations are modelled as white noise processes, while the bias variation is modelled as Brownian motion, i.e., random walk processes. These processes are defined in terms of Power Spectral Density (PSD) of the associated white noise processes and are tuned by the user to give the best navigation solution. The values used for processing this data are shown in Table 4.

Any observations, $z(t_{k})$, at discrete time instances, $t_{k}$, are included into the Kalman filter using a linear measurement model ([23] (Equations (4)–(136))):

(6) $$z\left(t_{k}\right)=H\left(t_{k}\right)\;x\left(t_{k}\right)+w_{m}\left(t_{k}\right),\;$$

where $H(t_{k})$ is the measurement matrix relating those observations to the system variables and $w_{m}\left(t_{k}\right)$ is a measurement noise vector. The observations are thus assumed to be a linear combination of state variables, corrupted by noise. The formation of such a measurement model for GNSS position and velocity observations is discussed in ([18] (Chapter 14.3)). From the GNSS and INS estimates, ${\hat{p}}_{k,\mathrm{GNSS}}=\left(\phi_{k,\mathrm{GNSS}},\lambda_{k,\mathrm{GNSS}},h_{k,\mathrm{GNSS}}\right)$, ${\hat{v}}_{k,\mathrm{GNSS}}^{n}$, ${\hat{p}}_{k,\mathrm{INS}}$ and ${\hat{v}}_{k,\mathrm{INS}}^{n}$, the measurement innovation is formed:

(7) $$\delta z_{k}\approx \left[\begin{array}{c} {\hat{p}}_{k,\mathrm{GNSS}}-{\hat{p}}_{k,\mathrm{INS}}\\ {\hat{v}}_{k,\mathrm{GNSS}}^{n}-{\hat{v}}_{k,\mathrm{INS}}^{n} \end{array}\right],\;$$

which is used to update the Kalman filter estimates as ([18] (Equations (3.24) and (3.61))):

(8) $${\hat{x}}_{k}={\hat{x}}_{k}^{-}+K_{k}\;\delta z_{k}\;\mathrm{and}\;P_{k}=P_{k}^{-}-K_{k}\left(H_{k}\;P_{k}^{-}\right),\;$$

where the superscript minus denotes values before the measurement update and $K_{k}$ is a weighting factor determining the influence of the new information. This factor is also known as the Kalman gain and is derived by minimising the trace of the updated error covariance matrix, $P_{k}$, i.e., minimising squared errors.

**Figure 3:**
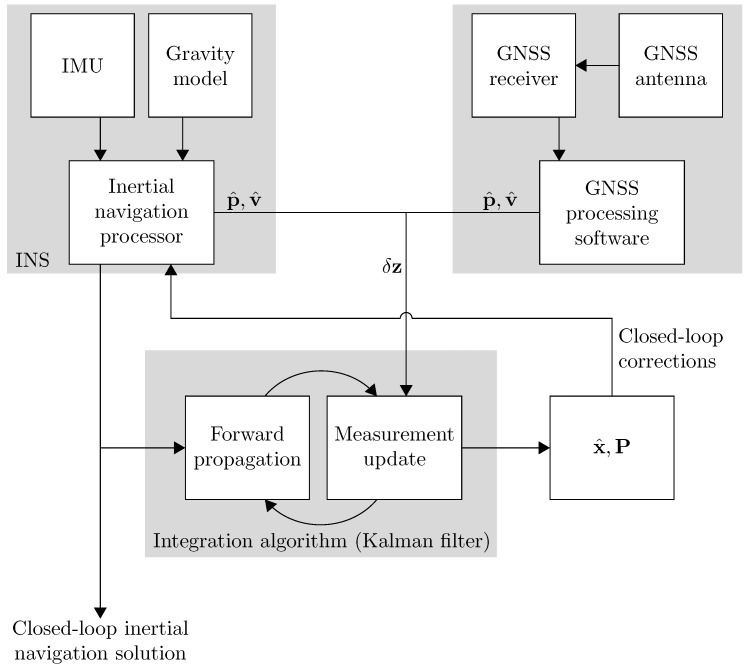
Overview of processing and data integration architecture.

The processing is thus performed using a semi-cascaded approach. First, the GNSS observations are processed into position and velocity estimates for the entire survey. Having initialised the INS solution, inertial navigation is performed until the next GNSS estimates are available. The INS solution is used to form a linear system model (4), which allows the propagation of the error covariance matrix forward in time, alongside the INS estimates. The stochastic error terms accounting for sensor errors will also influence this forward propagation. The INS and GNSS estimates are combined, using a least squares approach based on the associated error covariance, in order to yield statistically optimal estimates of the state variables. These optimal estimates are then used to correct the INS estimates, which are again propagated forward in time. This is illustrated in Figure 3.

The Kalman filter therefore has a cyclic nature, alternating between forward propagations and measurement updates. Since correlation between different state variables is built through the forward propagation phase, the Kalman filter also provides estimates of other states than those directly observed. In this way, the GNSS observations are not only used to correct the navigation solution, but also to continually calibrate the IMU, since estimates of sensor errors will be available.

Finally, in order to minimise linearisation errors and processing time, an error-state implementation was used in contrast to a total-state implementation. This means that the system model can be expressed as

(9) $$\left[\begin{array}{c} \delta \dot{p}\left(t\right)\\ \delta {\dot{v}}^{n}\left(t\right)\\ \delta {\dot{\psi }}_{nb}\left(t\right)\\ {\dot{b}}_{a}\left(t\right)\\ {\dot{b}}_{g}\left(t\right) \end{array}\right]=\left[\begin{array}{cccc} & 0_{3} & & 0_{3}\\ F_{\mathrm{INS}}\left(t\right) & C_{b}^{n} & & 0_{3}\\ & 0_{3} & & C_{b}^{n}\\ 0_{6\times 9} & & 0_{6} & \end{array}\right]\;\left[\begin{array}{c} \delta p(t)\\ \delta v^{n}\left(t\right)\\ \delta \psi_{nb}\left(t\right)\\ b_{a}\left(t\right)\\ b_{g}\left(t\right) \end{array}\right]+\left[\begin{array}{cccc} 0_{3} & 0_{3} & 0_{3} & 0_{3}\\ I_{3} & 0_{3} & 0_{3} & 0_{3}\\ 0_{3} & I_{3} & 0_{3} & 0_{3}\\ 0_{3} & 0_{3} & I_{3} & 0_{3}\\ 0_{3} & 0_{3} & 0_{3} & I_{3} \end{array}\right]\left[\begin{array}{c} w_{a}\left(t\right)\\ w_{g}\left(t\right)\\ w_{a,\mathrm{bias}}\left(t\right)\\ w_{g,\mathrm{bias}}\left(t\right) \end{array}\right]\;,$$

where δ denotes errors, $b_{a}$ are accelerometer biases and $b_{g}$ are gyroscope biases. The stochastic terms $w_{a}\left(t\right)$, $w_{g}\left(t\right)$, $w_{a,\mathrm{bias}}\left(t\right)$ and $w_{g,\mathrm{bias}}\left(t\right)$ are white noise processes defined in terms of their PSD. The form of the matrix, $F_{\mathrm{INS}}\left(t\right)$, is derived from the navigation equations by first applying a perturbation operator and then linearising with respect to the state variables ([19] (Chapter 5.4)).

### 3.3. Modelling Gravity as a Stochastic Process

Since the gravity model used for inertial navigation is not perfect, the gravity error (or disturbance) can be modelled as a stochastic process ([19] (Chapter 6.6)). A third-order exponentially time-correlated (Gauss–Markov) model is characterised by the autocorrelation function ([26] (Table 2.2-1)):

(10) $$R\left(\tau \right)=\sigma^{2}e^{-\beta |\tau |}\left(1+\beta |\tau |+\frac{1}{3}\beta^{2}{\left|\tau \right|}^{2}\right),\;$$

where σ is the standard deviation and β is a correlation parameter related to the correlation time as T=2.903/β. Since the Kalman filtering framework only allows the stochastic driving terms to be zero-mean white noise processes, the system model must be augmented as [27]:

(11) $$\begin{array}{c} \left[\begin{array}{c} \dot{p}\left(t\right)\\ {\dot{v}}^{n}\left(t\right)\\ {\dot{\psi }}_{nb}\left(t\right)\\ {\dot{b}}_{a}\left(t\right)\\ {\dot{b}}_{g}\left(t\right)\\ \frac{d}{dt}\delta g\left(t\right)\\ \frac{d}{dt}\delta \dot{g}\left(t\right)\\ \frac{d}{dt}\delta \ddot{g}\left(t\right) \end{array}\right]=\left[\begin{array}{cccccc} & 0_{3} & 0_{3} & 0_{3} & 0_{3} & 0_{3}\\ F_{\mathrm{INS}}\left(t\right) & C_{b}^{n} & 0_{3} & I_{3} & 0_{3} & 0_{3}\\ & 0_{3} & C_{b}^{n} & 0_{3} & 0_{3} & 0_{3}\\ & 0_{3} & 0_{3} & 0_{3} & 0_{3} & 0_{3}\\ & 0_{3} & 0_{3} & 0_{3} & 0_{3} & 0_{3}\\ 0_{15\times 9} & 0_{3} & 0_{3} & 0_{3} & I_{3} & 0_{3}\\ & 0_{3} & 0_{3} & 0_{3} & 0_{3} & I_{3}\\ & 0_{3} & 0_{3} & -\beta^{3}I_{3} & -3\beta^{2}I_{3} & -3\beta I_{3} \end{array}\right]\;\left[\begin{array}{c} p(t)\\ v^{n}\left(t\right)\\ \psi_{nb}\left(t\right)\\ b_{a}\left(t\right)\\ b_{g}\left(t\right)\\ \delta g(t)\\ \delta \dot{g}\left(t\right)\\ \delta \ddot{g} \end{array}\right]\\ \;+\left[\begin{array}{ccccc} 0_{3} & 0_{3} & 0_{3} & 0_{3} & 0_{3}\\ I_{3} & 0_{3} & 0_{3} & 0_{3} & 0_{3}\\ 0_{3} & I_{3} & 0_{3} & 0_{3} & 0_{3}\\ 0_{3} & 0_{3} & I_{3} & 0_{3} & 0_{3}\\ 0_{3} & 0_{3} & 0_{3} & I_{3} & 0_{3}\\ 0_{3} & 0_{3} & 0_{3} & 0_{3} & 0_{3}\\ 0_{3} & 0_{3} & 0_{3} & 0_{3} & 0_{3}\\ 0_{3} & 0_{3} & 0_{3} & 0_{3} & I_{3} \end{array}\right]\left[\begin{array}{c} w_{a}\left(t\right)\\ w_{g}\left(t\right)\\ w_{a,\mathrm{bias}}\left(t\right)\\ w_{g,\mathrm{bias}}\left(t\right)\\ w_{\mathrm{GM}3}\left(t\right) \end{array}\right],\; \end{array}$$

where the additional terms serve to “shape” the white noise into a Gauss–Markov process. The PSD amplitude of the associated white noise process, $w_{\mathrm{GM}3}\left(t\right)$, is related to the uncertainty and correlation parameters of the Gauss–Markov process as

(12) $$S_{\mathrm{GM}3}=\frac{16}{3}\;\sigma^{2}\;\beta^{5}=\frac{16}{3}\;\sigma^{2}\;{\left(|v_{\mathrm{hor}}|\;\beta^{\prime }\right)}^{5},\;$$

where the along-track correlation, $\beta =|v_{\mathrm{hor}}|\;\beta^{\prime }$, is specified in terms of distance instead of time and $v_{\mathrm{hor}}^{2}=v_{N}^{2}+v_{E}^{2}$ is the ground speed. This is because the gravity signal varies with position rather than time.

### 3.4. Introducing External Gravity Observations

External gravity observations can be introduced into the Kalman filter framework similar to position and velocity estimates, using a measurement model. For this flight test, we introduced observations from an A10 gravity meter at both JAV and KAGA stations. Although these observations represent only the magnitude of the gravity vector, the tie values are introduced as vector estimates

(13) $$z_{\delta g}={\left[\begin{array}{ccc} 0 & 0 & \delta g \end{array}\right]}^{⊤}\;\mathrm{with}\;R_{\delta g}\equiv \left[\begin{array}{ccc} 0.03 & 0 & 0\\ 0 & 0.03 & 0\\ 0 & 0 & 0.03 \end{array}\right],\;$$

where $R_{\delta g}$ is the associated error covariance matrix in units of mGal. The A10 measurement is corrected for the gravity model at the measurement location, meaning that the gravity disturbance is formed.

### 3.5. Smoothing

Instead of applying a regular filter, the Kalman filter estimates are smoothed by processing the data both forward and backward in time. The two solutions are then combined as

(14) $${\hat{x}}_{k}=P_{k}\;\left(P_{f,k}^{-1}\;{\hat{x}}_{f,k}+P_{b,k}^{-1}\;{\hat{x}}_{b,k}\right)\;\mathrm{and}\;P_{k}={\left(P_{f,k}^{-1}+P_{b,k}^{-1}\right)}^{-1},\;$$

where *f* denotes the forward solution and *b* the backward solution. This is accomplished in a processor-efficient way using the Rauch–Tung–Striebel (RTS) smoother ([26] (Chapter 5)).

## 4. Results

**Figure 4:**
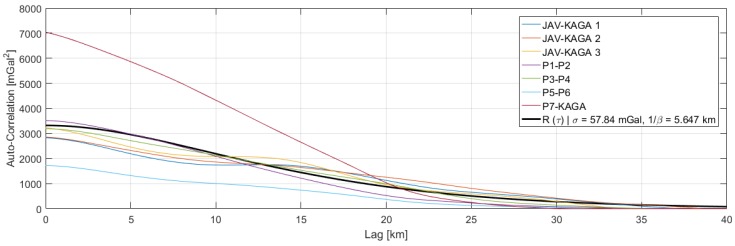
Estimated autocorrelation functions for the seven profiles along with the (least squares) best fitting third-order Gauss–Markov autocorrelation function. The parameters σ = 57.84 mGal and 1/β = 5.647 km.

Inertial navigation was performed using an implementation of Equation (1) and combined with GNSS velocity and position estimates using a Kalman filtering framework as described above. This resulted in an integrated IMU/GNSS solution, which was subsequently smoothed using an implementation of the RTS smoother. The gravity disturbance was modelled as a third-order Gauss–Markov process with initial parameters of σ = 100 mGal and β = 2.903/20 km. From the resulting gravity disturbance estimates, the autocorrelation function was estimated for each of the seven profiles (see Figure 4). A least squares fit of the Gauss–Markov autocorrelation function in Equation (10) yielded parameters of σ = 57.84 mGal and 1/β = 5.647 km, implying a correlation length of around 16 km, which was used for a second processing iteration.

### 4.1. Repeated Lines

**Figure 5:**
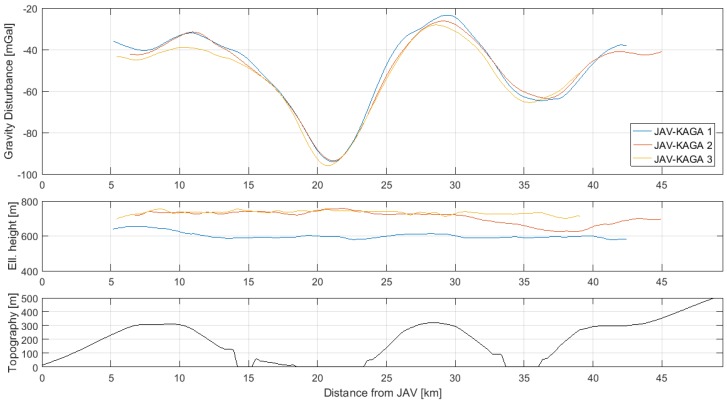
Profiles for the three repeated lines between JAV and KAGA. The gravity disturbance was modelled using a third-order Gauss–Markov model with uncertainty, σ = 57.84 mGal, and correlation, 1/β = 5.647 km: (**top**) gravity disturbance estimates; (**middle**) ellipsoidal height; (**bottom**) topography from SRTM30 with respect to WGS84 ellipsoid.

Gravity disturbance estimates for the three flights along the JAV-KAGA profile are shown in Figure 5, along with flight altitude and topography from the SRTM30 data product. The gravity variation clearly varies with topography, noticing that the areas around 15–25 km and 35 km are covered by ice.

The mean and standard deviation of the differences between the three profiles are shown in Table 5. Since the long wavelength information is assumed to be corrupted by sensor error variation, the profiles are corrected for a bias and linear trend, before the differences are formed.

### 4.2. Comparison with AIRGrav Profiles

**Figure 6:**
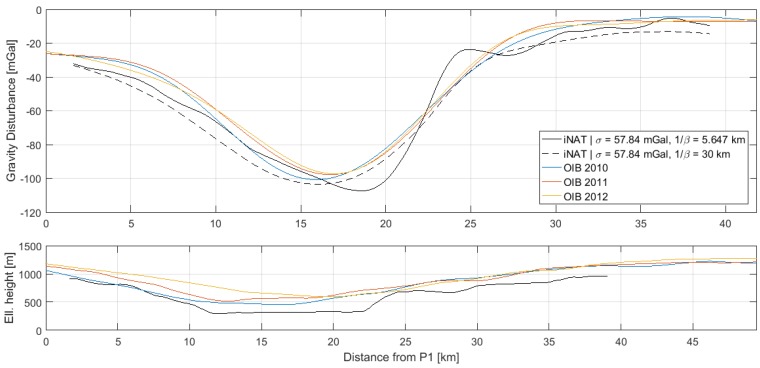
Gravity disturbance estimates for the profile between P1 and P2 for two different Gauss–Markov correlation lengths: (**top**) gravity disturbance profile; (**bottom**) height profile.

**Figure 7:**
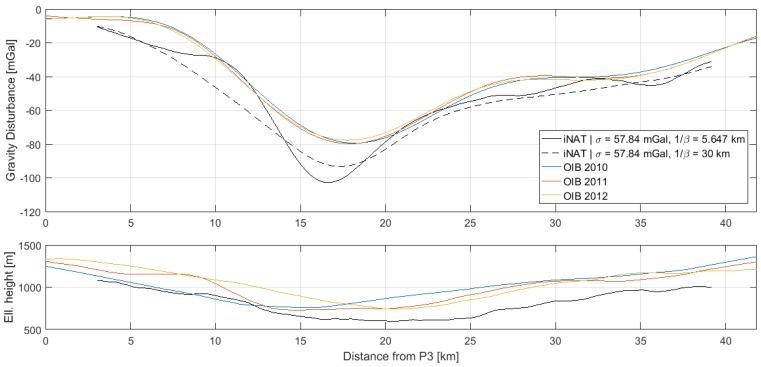
Gravity disturbance estimates for the profile between P3 and P4 for two different Gauss–Markov correlation lengths: (**top**) gravity disturbance profile; (**bottom**) height profile.

**Figure 8:**
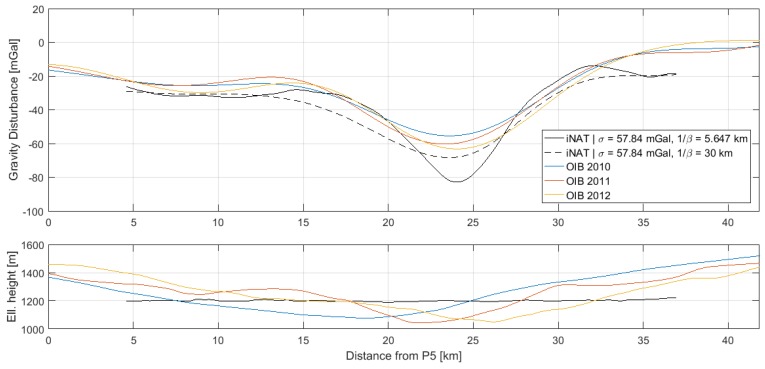
Gravity disturbance estimates for the profile between P5 and P6 for two different Gauss–Markov correlation lengths: (**top**) gravity disturbance profile; (**bottom**) height profile.

Gravity disturbance estimates along the three profiles, P1–P2, P3–P4 and P5–P6, are shown in Figure 6, Figure 7 and Figure 8, along with the AIRGrav gravity profiles from OIB. From the height profiles, it is evident that the lines P1–P2 and P3–P4 were flown in draped mode (following the topography). The first line closely follows the terrain, whereas the second line is more smooth. The third line, P5–P6, was flown at constant altitude. The OIB lines were also flown in draped mode. Since the gravity signal attenuates with distance from source, the elevation will influence the spatial resolution of the gravity profile. However, aircraft dynamics will influence the observed signal and may therefore also have an impact on the recovered gravity signal.

The AIRGrav instrument was carried by a fixed-wing aircraft flying at approximately 134 m/s, whereas the iNAT instrument was flown in a helicopter at approximately 52 m/s. Therefore, the iNAT gravity profiles have a higher spatial resolution than the OIB profiles. This is also evident from the figures, indicating that the glacier gravity anomaly is more sharp than indicated by the OIB profiles. Since the correlation length of the Gauss–Markov stochastic process will influence the resolution of the gravity profile, it was increased in order to arrive at gravity profiles with a resolution similar to that of the OIB estimates. The optimal similarity in terms of Root-Mean-Square (RMS) difference occurs around 1/β=30 km. The resulting profiles are also shown in the above figures and the statistics of the differences are shown in Table 6.

### 4.3. Cross-Over Evaluation

**Figure 9:**
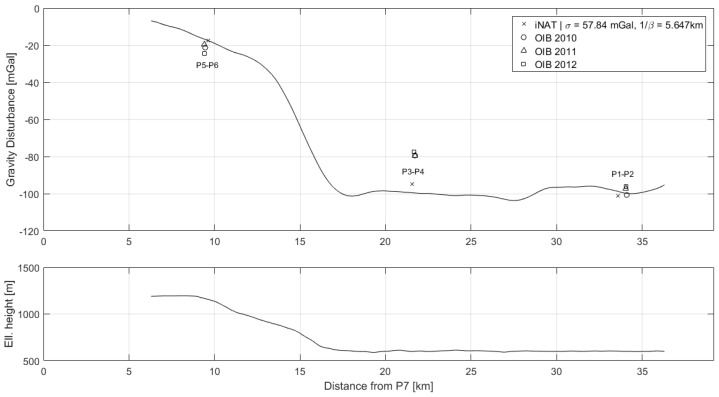
Profile along the P7-KAGA line which crosses over the three OIB lines: (**top**) gravity disturbance profile along with values from crossing lines; (**bottom**) height profile.

The gravity profile along the P7-KAGA line is shown in Figure 9 together with the gravity estimates from the crossing lines. This line was flown at constant altitude over the glacier before increasing in altitude to reach the KAGA site. The cross over differences are listed in Table 7.

## 5. Discussion

The statistics from the repeated lines in Table 5 suggest that the gravity estimates are biased with respect to one another. As argued in the introduction, the temperature variation is suspected to corrupt the long wavelength information in the gravity estimates. The first two profiles are least biased and are flown consecutively at the beginning of the survey. The third profile, which has a larger bias, was flown at the end of the survey, where the sensor errors have had time to evolve. With the mean value removed, the agreement between the profiles are 2.00–2.73 mGal and, by additionally removing a linear trend, the agreement improves to 1.95–2.38 mGal. The convention for comparing airborne gravity estimates is in terms of the Root-Mean-Square-Error (RMSE), which is related to the standard deviation as

(15) $$\sigma =\sqrt{\frac{\sum {\left(x-\bar{x}\right)}^{2}}{N-1}}\;,\;RMS=\sqrt{\frac{\sum x^{2}}{N}}\;\mathrm{and}\;RMSE=RMS/\sqrt{2}\;,$$

meaning that the accuracy is better than 2 mGal. The cross over differences from Table 7 do also fit within this confidence level.

Since the RTS smoother does not have an associated filter length, it is not straightforward to associate the profiles with a spatial resolution. This is, however, an important issue, if the results are to be compared with other studies. In Figure 6, Figure 7 and Figure 8, it was shown how the correlation length of the Gauss–Markov process could be varied to effectively control the degree of smoothing applied to the gravity profile. However, since the standard deviation of the process will also influence the degree of smoothing, the connection between correlation length and equivalent filter length is also not straightforward. Moreover, the parameters of the stochastic model will influence the Kalman filter estimates themselves and not only the RTS smoothed estimates, further complicating the issue.

**Figure 10:**
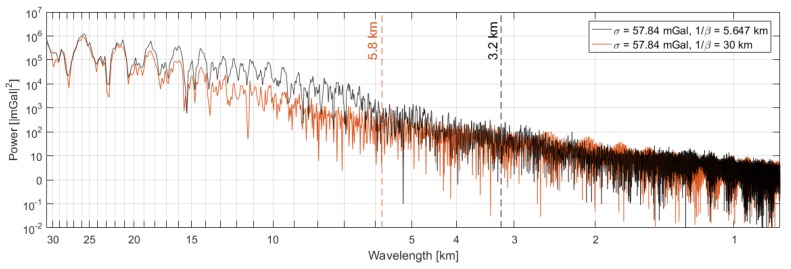
Power spectrum of the estimated gravity disturbance across the entire flight. The spectrum is also shown for the over-smoothed estimates and the estimated spatial resolutions are indicated by vertical lines.

In order to estimate the equivalent filter length and thus the spatial resolution of the gravity estimates, a set of alternative gravity estimates, independent of any stochastic model, was derived from the data. From Equation (1), the specific force, $f^{b}$, observed by the IMU is related to gravity as

(16) $$g^{n}={\dot{v}}^{n}-\left(C_{b}^{n}\;f^{b}-\left(2\Omega_{ie}^{n}+\Omega_{en}^{n}\right)\;v^{n}\right),\;$$

where the term inside the parenthesis can be formed using the RTS smoothed Kalman filter estimates. The observed specific force is corrected for bias variation, resolved about the *n*-frame axes and corrected for the velocity dependent fictitious forces. The term, $\dot{v}{,}^{n}$ can be derived from the GNSS position solution using a 2nd order central difference approach. Gravity estimates are derived by differencing these two components and applying a two-pass Butterworth filter. By tuning the filter length to match the Kalman filter gravity estimates, an estimate of the equivalent filter length is available. This was done on a line-to-line basis. Then, using the average ground speed along each line, the filter length was converted to a (half-wavelength) spatial resolution. The average spatial resolution for the seven lines is 3.2 km with a standard deviation of 1.1 km. The power spectrum for the entire survey is shown in Figure 10.

Due to the significant variation in estimated spatial resolution, a conservative estimate is 4.5 km, where the power spectrum reaches the $10^{2}$ magnitude level. Also shown in the figure is the power spectrum for the over-smoothed solution, i.e., with 1/β=30 km, containing less power in the 6–20 km wavelength interval. Using the same approach as before, the spatial resolution was estimated to 5.8 km with a standard deviation of 1.1 km. Since these estimates were tuned to mimic the AIRGrav OIB profiles, the expected spatial resolution is 6.7 km, which is within the confidence bounds. Some inconsistency may also originate from the filter, since the shape of the filter applied in the AIRGrav processing is unknown to the authors.

This approach does, however, not seem to form a consistent connection between spatial resolution in terms of a filter and in terms of a stochastic model. As this connection is important in order to compare results, further investigations are encouraged.

## 6. Conclusions

It has been shown that strapdown airborne gravimetry is feasible from a helicopter platform. The strapdown system is attractive since it is easily installed and does not require any operation during the flight. Moreover, no signs of degradation were seen during draped flying. With mean values removed, the accuracy was estimated to better than 2 mGal at a half-wavelength spatial resolution of 4.5 km.

It was also found that the fixed-wing OIB profiles across the Jakobshavn Glacier underestimated the peak gravity anomaly by up to 20 mGal as a consequence of the faster aircraft speed.

## Figures and Tables

**Table 1:** Accelerometer drift over the entire flight.

	*x*-axis	*y*-axis	*z*-axis	
Total drift				
- without calibration	1.730	−1.192	−8.422	mGal
- with calibration	1.419	−0.820	3.496	mGal
Drift rate				
- without calibration	391.8	−269.8	−1907	μGal/h
- with calibration	321.3	−185.7	791.8	μGal/h

**Table 2:** Mean and standard deviation (STD) of the difference in gravity estimates (100 s full wavelength filtering) along the three Operation IceBridge (OIB) lines.

		2010–2011	2010–2012	2011–2012	
**P1–P2**	Mean	1.03	1.04	−0.13	mGal
	STD	2.66	2.93	1.65	mGal
**P3–P4**	Mean	0.65	0.44	−0.20	mGal
	STD	1.84	1.82	1.43	mGal
**P5–P6**	Mean	−0.17	−1.06	−0.95	mGal
	STD	2.70	3.19	3.74	mGal

**Table 3:** Overview of relevant reference frames.

Frame	Origin	*z*-axis	*x*-axis	*y*-axis
*i*	Earth centre of mass	Earth rotational axis	Equatorial plane, vernal equinox	Completes right-handed system
*e*	Earth centre of mass	Earth rotational axis	Equatorial plane, Greenwich meridian	Completes right-handed system
*n*	Instrument location	Down along ellipsoidal normal	North	East
*b*	Instrument location	Through-the-floor (down)	Forward	Starboard (right)

**Table 4:** Stochastic models and amplitude of the associated Power Spectral Densities (PSDs).

	Error Model	PSD Amplitude	
Accelerometer noise	White noise	0.05	$\mathrm{mm}/s/\sqrt{s}$
Gyroscope noise	White noise	0.2	$\mathrm{arcsec}/\sqrt{s}$
Accelerometer bias variation	Random walk	0.01	$\mathrm{mGal}/\sqrt{s}$
Gyroscope bias variation	Random walk	3.0 × $10^{-5}$	${}^{\circ }/h/\sqrt{s}$

**Table 5:** Mean and standard deviation of the difference in gravity estimates for the three repeated lines along the JAV-KAGA profile. The difference was formed from the profiles directly and by first removing a bias and linear trend from each profile.

		No Correction	Bias + Trend	
**Lines 1–2**	Mean	−0.83	0	mGal
	STD	2.00	1.95	mGal
**Lines 1–3**	Mean	−3.25	0	mGal
	STD	2.73	2.38	mGal
**Lines 2–3**	Mean	−3.31	0	mGal
	STD	2.28	2.15	mGal

**Table 6:** Mean and standard deviation of the difference in gravity estimates along the three profiles. The gravity estimates were smoothed by increasing the correlation parameter to 1/β=30 km.

		No Correction			Bias + Trend			
		2010	2011		2010	2011	2012	
**P1–P2**	Mean	7.10	8.86	8.46	0	0	0	mGal
	STD	3.79	4.90	4.20	3.59	4.20	3.70	mGal
**P3–P4**	Mean	10.8	10.4	10.5	0	0	0	mGal
	STD	4.96	4.42	4.19	3.84	3.51	2.83	mGal
**P5–P6**	Mean	8.46	8.04	6.07	0	0	0	mGal
	STD	3.40	2.96	5.28	3.16	2.91	5.22	mGal

**Table 7:** Difference in gravity disturbance at the intersection points between the P7-KAGA line and the three OIB lines.

	iNAT	OIB 2010	OIB 2011	OIB 2011	
**P1–P2**	2.36	0.85	2.56	3.61	mGal
**P3–P4**	4.83	20.2	20.0	22.2	mGal
**P5–P6**	0.07	4.42	2.98	7.78	mGal
